# Supplementary material for: Calcitonin Related Polypeptide Alpha Mediates Oral Cancer Pain
Source: Cells. 2023 Jun 21;12(13):1675. doi: 10.3390/cells12131675 (PMC10341289; doi:10.3390/cells12131675)
Supplement: Supplementary file 1 [file cells-12-01675-s001.zip › Supplementary_Figures_S1-S4.pdf]

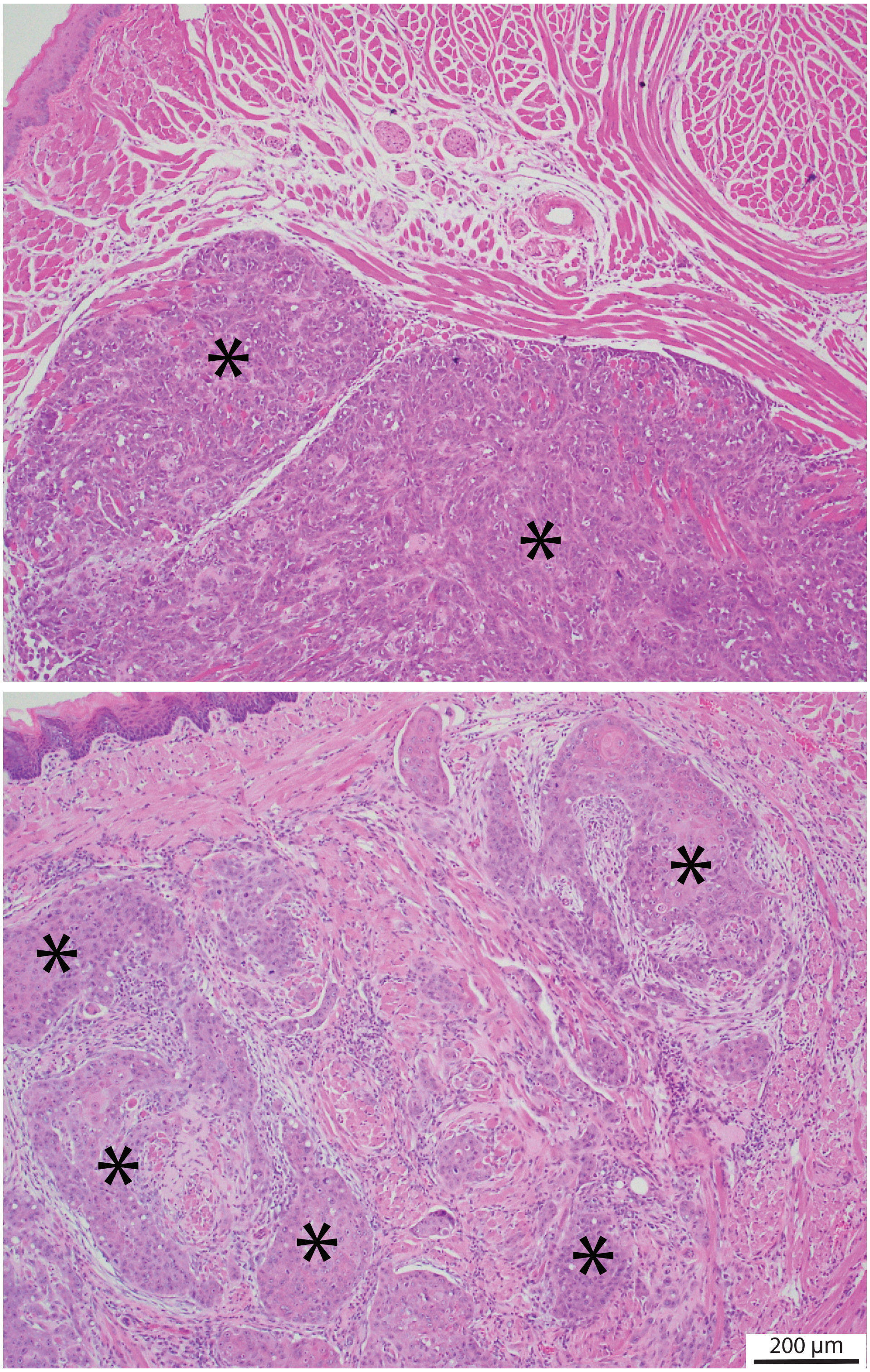

Figure S1. Growth phenotype of HSC-3 (top panel) and OSC-20 (bottom panel) orthotopic xenografts. HSC-3 tumors (top) grow as compact masses that displace the tongue musculature. OSC-20 tumors (bottom) are characterized by tumor islands with prominent stroma. Asterisks indicate tumor cells. Magnification of both photomicrographs is shown in the bottom panel.

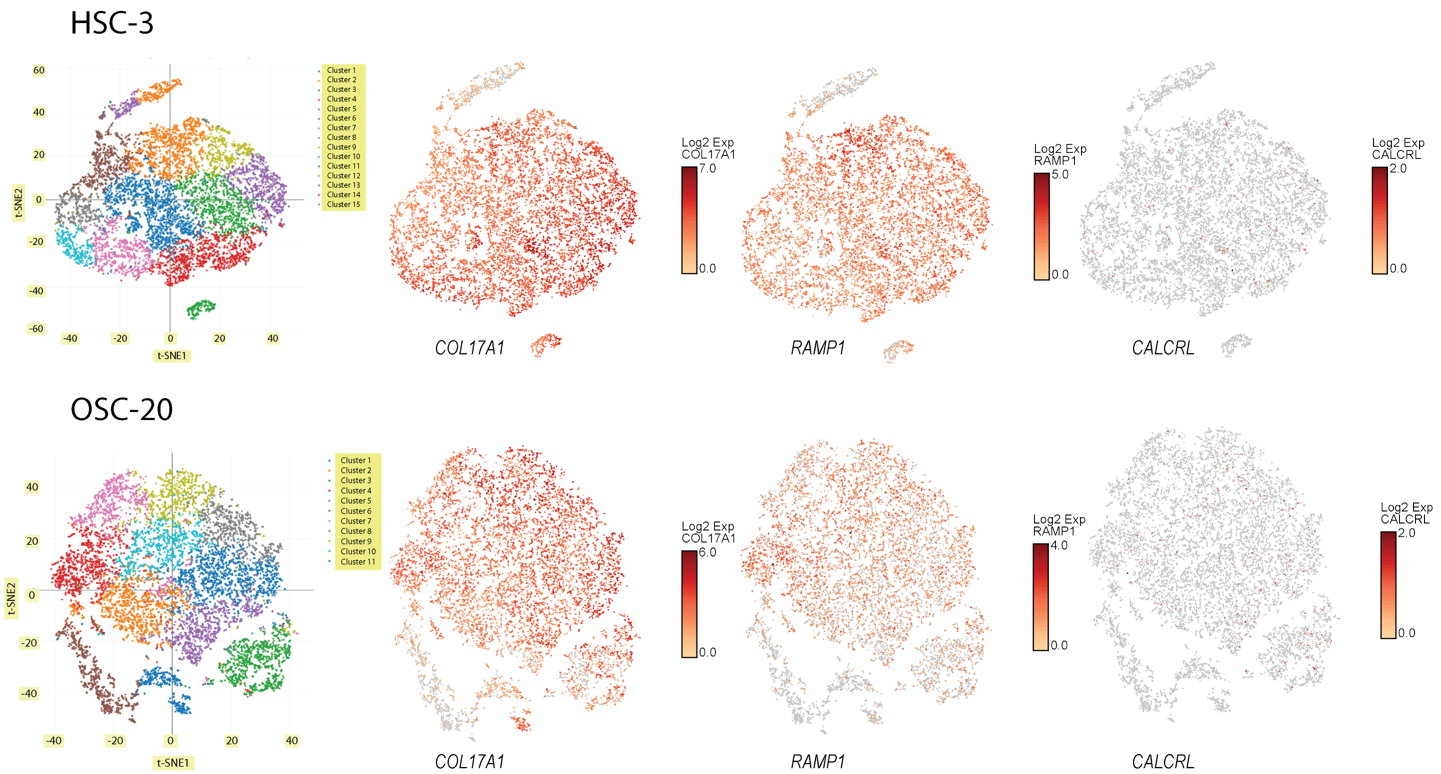

Figure S2. Expression of *RAMP1*, *CALCRL* and *COL17A1* in HSC-3 and OSC-20 cells grown on tissue culture plastic. Expression profiles of selected genes are indicated on the t-SNE representations of HSC-3 and OSC-20 cells.

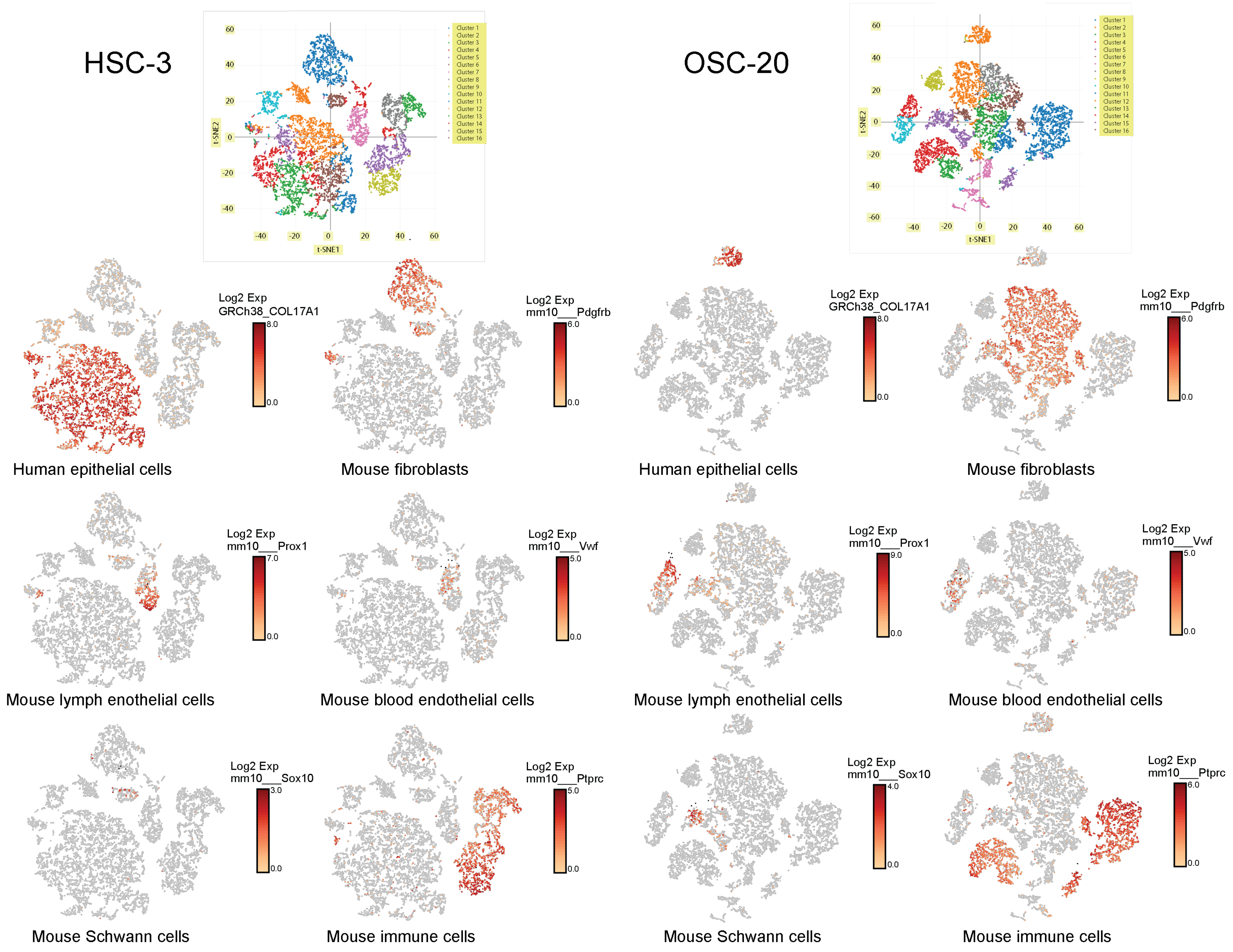

Figure S3. Heatmap showing expression of marker genes for immune cells (*Ptprc*, encoding CD45), fibroblasts (*Pdgfrb*), lymph endothelial cells (*Prox1*, *Lyve1*), blood endothelial cells (*Vwf*) and Schwann cells (*Plp1*). Expression profiles are indicated on the t-SNE representations of HSC-3 and OSC-20 xenografts.

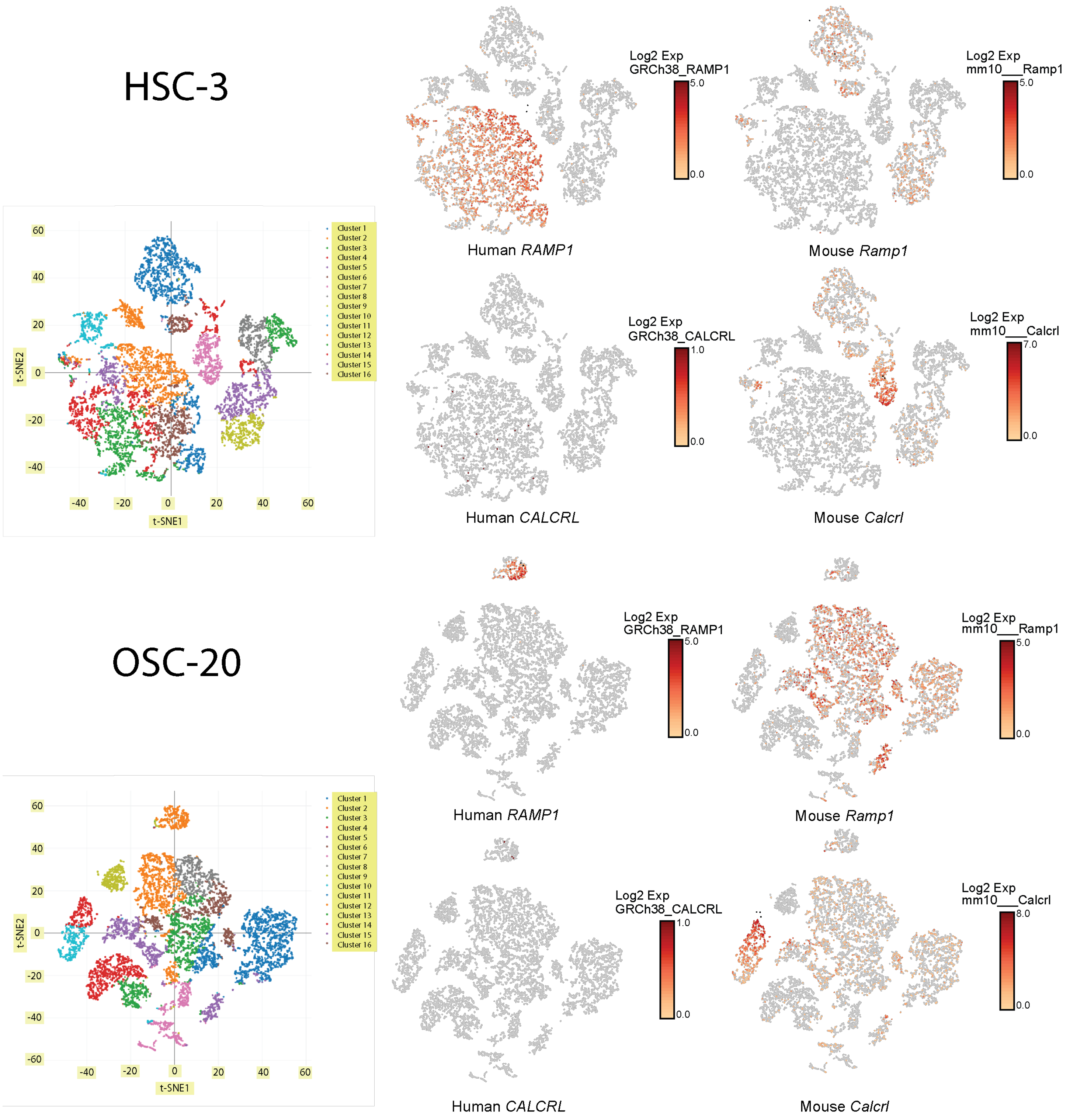

Figure S4. Heatmap showing expression of human and mouse CGRP receptor components in HSC-3 and OSC-20 xenografts. Expression profiles are indicated on the t-SNE representations of HSC-3 and OSC-20 xenografts.
