# Supplementary material for: Calcitonin Related Polypeptide Alpha Mediates Oral Cancer Pain
Source: Cells. 2023 Jun 21;12(13):1675. doi: 10.3390/cells12131675 (PMC10341289; doi:10.3390/cells12131675)
Supplement: Supplementary file 1 [file cells-12-01675-s001.zip › Supplementary_Table_1.pdf]

Table S1. Antibodies for multiplex immunofluorescence.

Targe/1° Ab	Vendor/ Cat #	Research Resource Identification	Clone	Heat retrieval	Dilution	1° Ab Incubation time (min)	OpalFluor	Vendor/ Cat#	Dilution
CALCA	LsBio, LS-C661894	Not assigned	poly	none	100	30	570	Akoya FP1488001KT	150
SOX10	Sigma 383R-14	Not assigned	EP268	ER2-20	100	30	690	Akoya FP1497001KT	150
LYVE1	Abcam ab218535	Abcam Cat# ab218535, RRID:AB_2927473	EPR21771	ER2-20	4000	30	520	Akoya FP1487001KT	150
GAP43	Novus, NB300143	Novus Cat# NB 300-143, RRID:AB_350468	Poly	ER2-20	4000	30	480	Akoya FP1500001KT	150
CD31, PECAM	CST, 77699	Cell Signaling Technology Cat# 77699, RRID:AB_2722705	D8V9E	ER2-20	100	30	620	Akoya FP1495001KT	150
p40/	Abcam, ab203826	Abcam Cat# ab203826, RRID:AB_2934266	EPR17863-47	ER2-20	250/	30	780	Akoya FP1501001KT TSA-DIG & OP780	150
p63 cocktail	Abcam, ab124762	Abcam Cat# ab124762, RRID:AB_10971840	ERP5701		4,000				

All Ab's were diluted in Leica diluent (#AR9352) except CD31 which was diluted in CST SignalStain diluent (#8112L). The polymer and Opal fluorophor incubation times were 10 mins each.
